# Simultaneous closure of a perilymphatic fistula and placement of cochlear implant in a case of complex inner ear malformation

**DOI:** 10.1002/ccr3.9423

**Published:** 2024-09-03

**Authors:** Hanan Almuzaini, J. Müller, Flatz Wilhelm, D. Polterauer, M. Schuster

**Affiliations:** ^1^ General and Specialized Surgery Department Medicine College, Taibah University Madinah, KS Saudi Arabia; ^2^ Department of Otorhinolaryngology, Head and Neck Surgery Ludwig‐Maximilians‐University of Munich Munich Germany; ^3^ Department of Radiology Ludwig‐Maximilians‐University of Munich Munich Germany

**Keywords:** cochlea implantation, developmental delay, inner ear malformation, meningitis, perilymphatic fistula

## Abstract

**Key Clinical Message:**

In young infants, under the age of one‐year, cochlear malformation with profound hearing loss complicated by a perilymphatic fistula (PLF), presents a serious clinical challenge, warranting immediate audiological and surgical intervention. Timely PLF detection and closure, along with an early CI can significantly improve the prognosis of such patients and helps them in achieving their maximum hearing and developmental potential, in the long term.

**Abstract:**

Inner ear malformation (IEM) with incomplete partition and cystic cochlea is mostly accompanied by profound hearing loss. It gets further complicated with other malformations such as a perilymphatic fistula (PLF). This case concerns an 8‐month‐old child cochlear malformation and profound hearing loss. Surgical intervention identified a PLF at the stapedial footplate, which was successfully closed. The surgery also included the placement of a cochlear implant (CI) in the right ear, via the round window. The left ear was equipped with hearing aids, with persistent hearing thresholds at 70–80 db. At the age of 6 years, the child showed a good hearing outcome with the CI, with only moderate speech delay. Cochlear malformation accompanied by a perilymphatic leakage warrants immediate surgical closure of the PLF, to minimize the risk of bacterial meningitis. Wherever possible, the feasibility of a CI should be explored in such cases and a CI should be placed for treatment of hearing loss. Audiological and speech outcomes may vary with the use of the CI, especially in cases of IEM. However, an early CI coupled with timely PLF detection and closure can help children with profound hearing loss, in achieving their maximum hearing and developmental potential, in the long run.

## 1 INTRODUCTION

An estimated 20%–30% of congenital hearing loss in children is due to inner ear malformation (IEM).^1^ The IEMs are divided into two categories that differentiate patients with cochlear malformation or aplasia, from those having a normal cochlea with an abnormal vestibule or semicircular canals. In 1987, Jackler et al, classified this malformation into five types containing complete labyrinthine aplasia, cochlear aplasia, cochlear hypoplasia, incomplete partition (IP), and common cavity.^2^ Sennaroglu introduced a more detailed system by classifying IP into two subtypes: IP‐I (less than 1.5 turns) and IP‐II (between 1.5 and 2.75 turns, or a classic Mondini deformity).^1^

Sometimes, cochlear malformation is part of a more complex deformation that might cause perilymphatic leakage.^3^, ^4^, ^5^ Both, cochlear malformation and perilymphatic fistula (PLF) can lead to hearing loss as well as bacterial dissemination from the middle ear to the cerebrospinal fluid (CSF). In such cases, bacterial meningitis is a known complication and can lead to quick, progressive inner ear hearing loss.^6^, ^7^

In a complex IEM with severely changed morphology and shortened cochlea, cochlear implantation (CI) is challenging or sometimes impossible to perform. The reported outcomes are not quite encouraging, often leading to poor speech perception and language development.^8^ In this article, we report the rare case of an 8‐month‐old child with severe cochlea malformation and perilymphatic leakage, successfully managed by simultaneous closure of a PLF, combined with CI.

## 2 CASE PRESENTATION

An 8‐month‐old girl with a prior history of failed otoacoustic emission (OAE) and automated auditory brainstem response (AABR) tests was admitted to the Department of Pediatric Audiology, with a clinical suspicion of hearing loss. The child also had a history of generalized mild motor developmental delay. At the age of 7 months, she was treated for pneumococcal meningitis and showed good general recovery.

## 3 METHODS (INVESTIGATION, DIAGNOSIS, AND SURGICAL MANAGEMENT)

Initial clinical examination showed well‐ventilated middle ears, confirmed by tympanometry showing a Type A tympanogram. Behavioral audiometry revealed moderate to severe hearing loss and missing transient evoked otoacoustic emission (TEOAE) and distortion product otoacoustic emissions (DPOAE) bilaterally.

**FIGURE 1:**
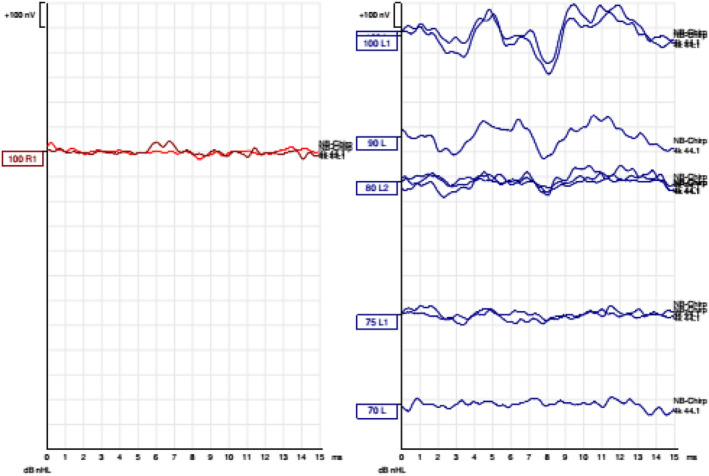
Frequency‐dependent ABR Results* of stimulation using NB‐chirp‐stimuli. ABR, auditory brainstem response; N, narrow band.

As the clinical presentation and investigations raised the suspicion of bilateral permanent hearing loss, the child underwent general anesthesia for the purpose of detailed audiological assessment. Microscopy of the right ear revealed middle ear effusion; myringotomy was performed to drain the effusion and relieve the child. This was followed by ABR and auditory steady‐state response (ASSR) assessments performed on both ears, (Interacoustics Eclipse EP25, Denmark) with Ear Tone ABR3A transducers (earplugs) and contralateral noising. Using clicks for air and bone conduction, no threshold could be detected below 100 dB or 45 dB, respectively, in both ears. Frequency‐dependent ABR using narrow band chirps with center frequencies at 500 Hz, 1 kHz, 2 kHz, and 4 kHz, revealed no responses in the right ear at a maximum stimulus of 100 dB. In the left ear no response could be elicited at 1 KHz. However, a response was obtained at 2KHz at 70 dB and 4KHz at 80 dB. ASSR at center frequencies of 500 Hz, 1 kHz, 2 kHz, and 4 kHz showed no responses in the right ear, with a maximal stimulus level of 90 dB at each frequency range, and in the left ear at 70 dB, 80 dB, 70 dB, 70 dB (Figure 1).

**FIGURE 2:**
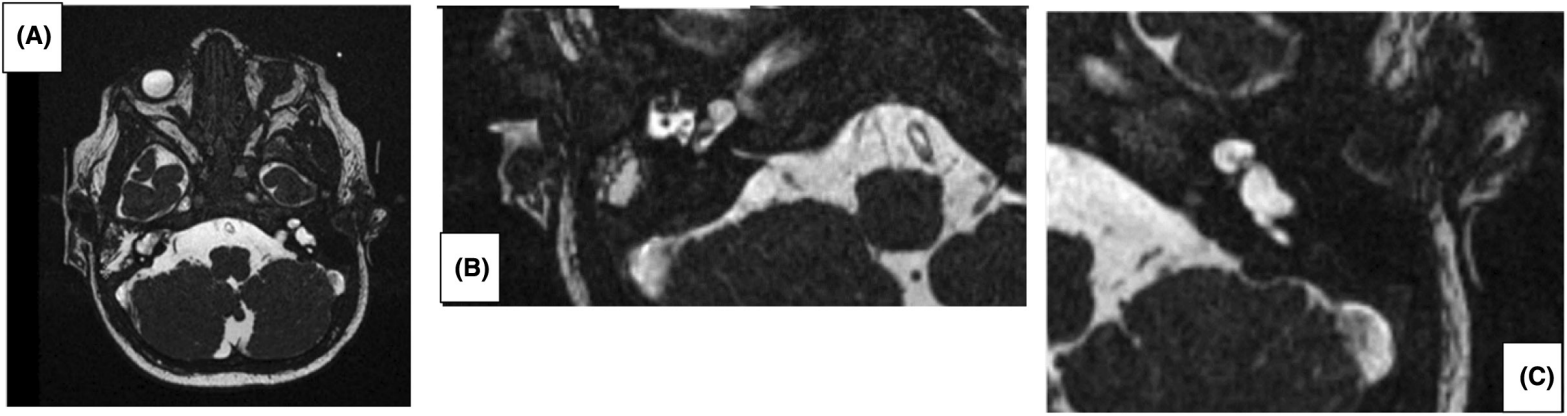
(A) MRI of the inner ear showing a malformation of both inner ears with detailed view of the right (B) and left ear (C). MRI, magnetic resonance imaging.

Subsequently, the patient underwent a magnetic resonance imaging (MRI) scan of the head with high resolution imaging of the inner ear, while yet under general anesthesia. It revealed a malformation of both inner ears (Figure 2A). On the right ear, a common cavity deformity (CCD) was seen (Figure 2B). A malformation was seen on the border in between IP‐I and CCD in the left ear (Figure 2C). The 8th cranial nerve on both sides, showed a clear separation into cochlear and vestibular branches, in both ears. (Figure 2A).

**FIGURE 3:**
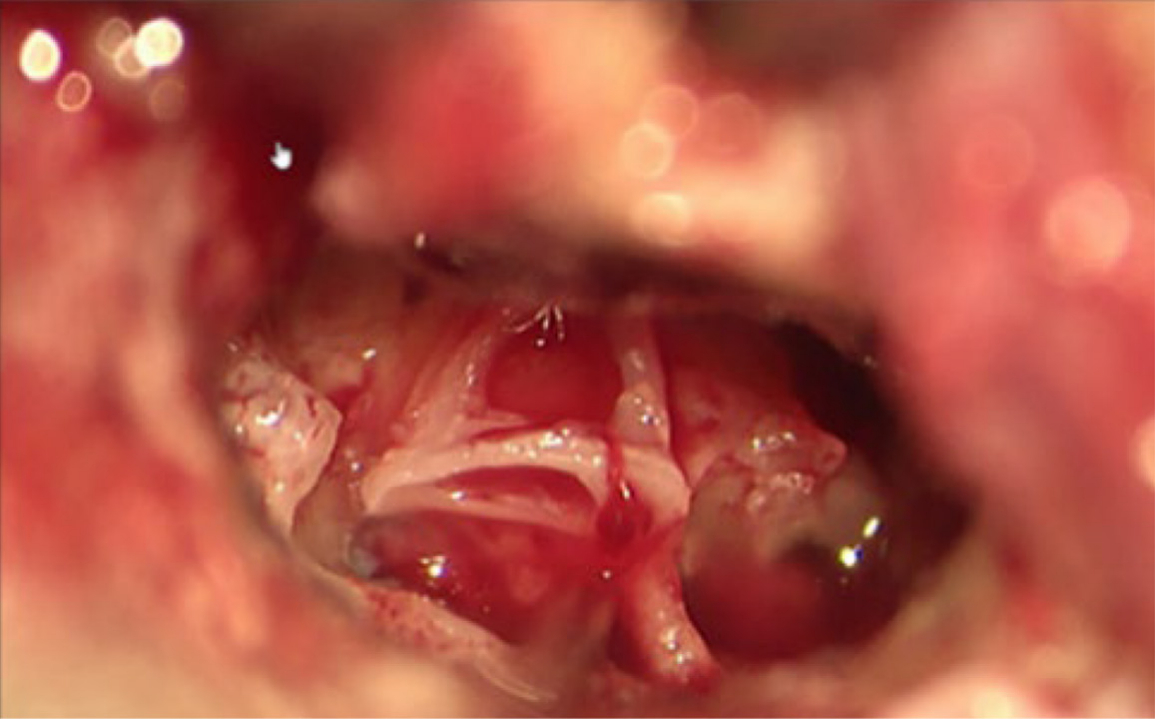
Perilymphatic leakage identified at the oval window.

**FIGURE 4:**
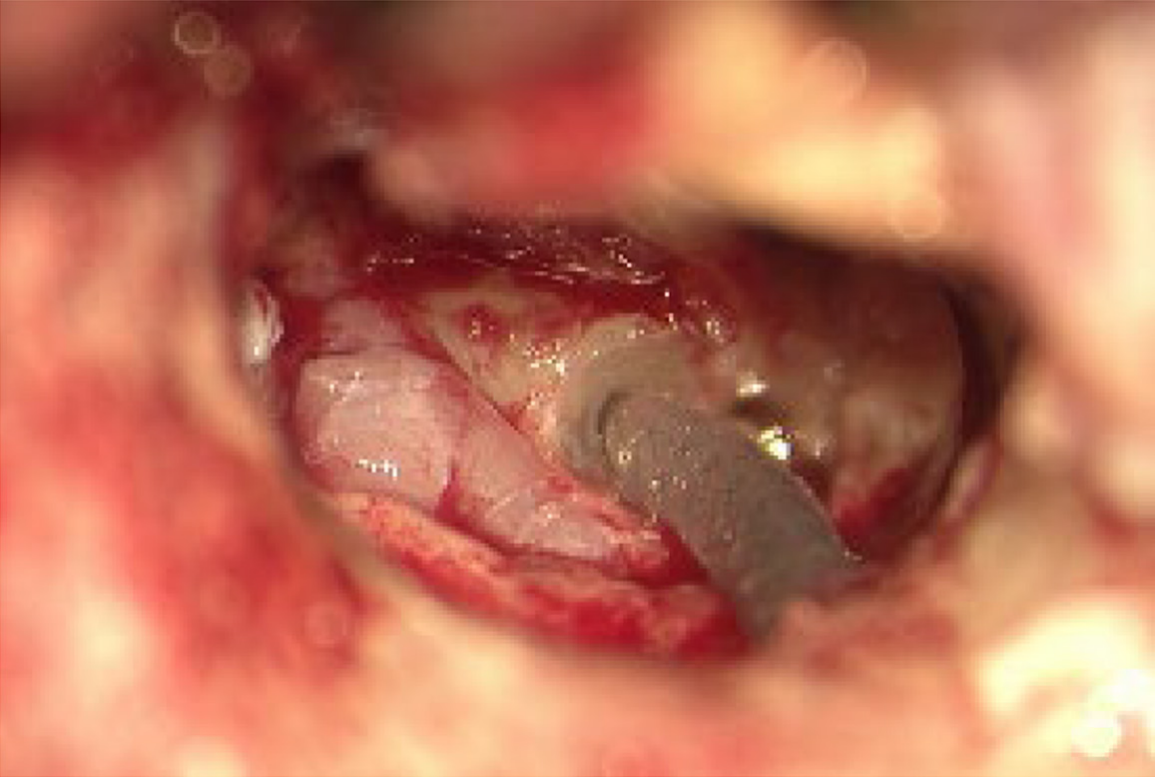
Surgical site after implantation of a 28 mm‐electrode via the round window.

**FIGURE 5:**
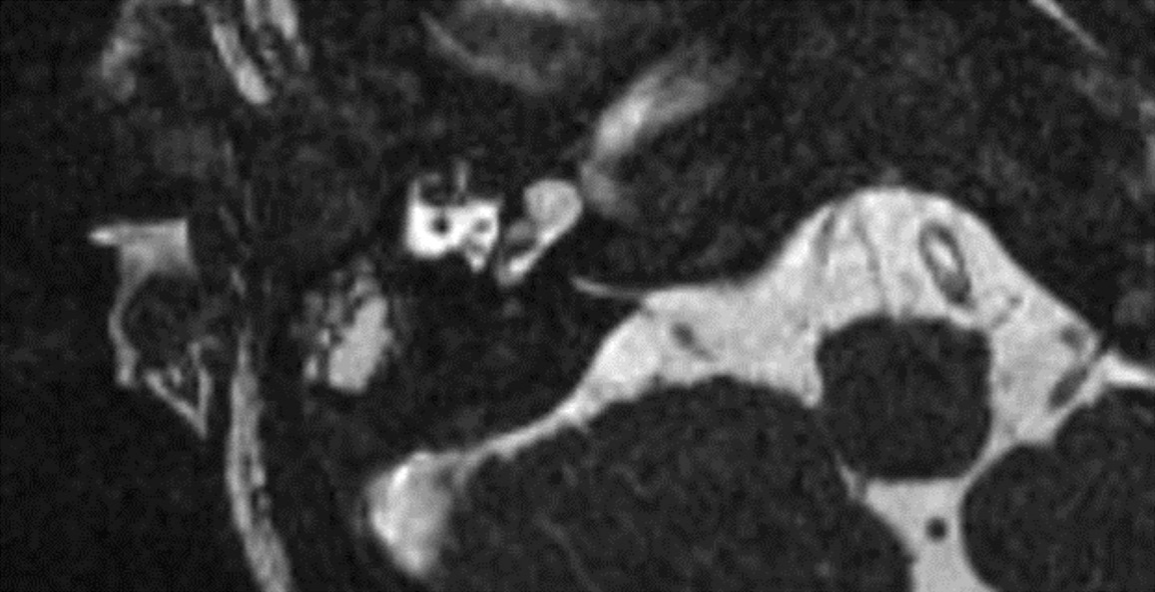
Postoperative CT scan of the cochlea showing the localization of the implant.

A couple of days later, following myringotomy, there was constant clear otorrhea from the right ear. A biochemical test for β2 transferrin confirmed the presence of CSF leak in the right middle ear, which indicated a PLF, with an obviously associated risk of meningitis. Therefore, surgical treatment was scheduled immediately. Surgery included a mastoidectomy with obliteration of the PLF, followed by placement of a CI on the right side. The fistula was identified as a perilymphatic leak under the stapes footplate, at the oval window (Figure 3). It was closed using connective tissue, including fascia from the temporal muscle, after partial removal of the footplate. A non‐preformed 28 mm electrode was chosen due to the altered anatomy (Figure 4). Postoperative CT scan confirmed appropriate localization of the electrode. (Figure 5).

## 4 OUTCOME AND FOLLOW‐UP

After closure of the PLF and placement of the CI, there was no further otorrhea, rhinorrhea or signs of any fluid in the right middle ear. On the left ear, hearing amplification was successfully adapted to the measured hearing threshold.

Postoperative CI rehabilitation began 6 weeks after surgery with regular mapping and fitting of the implant. On the left ear, hearing threshold and hearing amplification were controlled. On regular follow‐up, clinical examinations and investigations did not show any recurrence of otorrhea or meningitis. At the one‐year follow‐up visit, behavioral audiometry on the right ear using the CI showed hearing level thresholds ranging between 40–45 dB at 0, 250–6 KHz and at 30 dB with a frequency of 8 KHz. In the left ear, the hearing aid showed hearing level thresholds between 45 and 55 dB in all measured frequencies. Speech and language evaluation revealed delayed development. The child spoke six words purposefully. Speech comprehension clearly developed in both languages; her mother language is Turkish and her second language is Deutsche. In daily life, she would respond well to soft speech or whispering, but had delayed reactions in noisy situations. She continued to receive regular speech therapy.

The follow‐up at the age of 3 years showed the following results: the right tympanic membrane was slightly scarred, and the left tympanic membrane was intact, both with a well‐ventilated middle ear. Tympanometry revealed Type A on both sides. On the right side having the CI, visual enforced audiometry, showed hearing thresholds ranging between 50 and 65 dB at 500 Hz–4KHz, in the left ear, the hearing aid showed a hearing threshold of 60 dB at 250 Hz‐4 KHz. Telemetry of the CI revealed a good outcome. The functions of the microphone, sound processor, internal implant and the electrode were satisfactory. The speech processor of the CI had been adjusted slightly. Speech evaluation revealed delayed development with restricted vocabulary, morpho‐syntactic and phonetic abilities. She produced 2–3 word‐utterances; Her pronunciation was understandable to her family. The patient was advised to continue speech therapy and regular follow‐up every 6 months.

**FIGURE 6:**
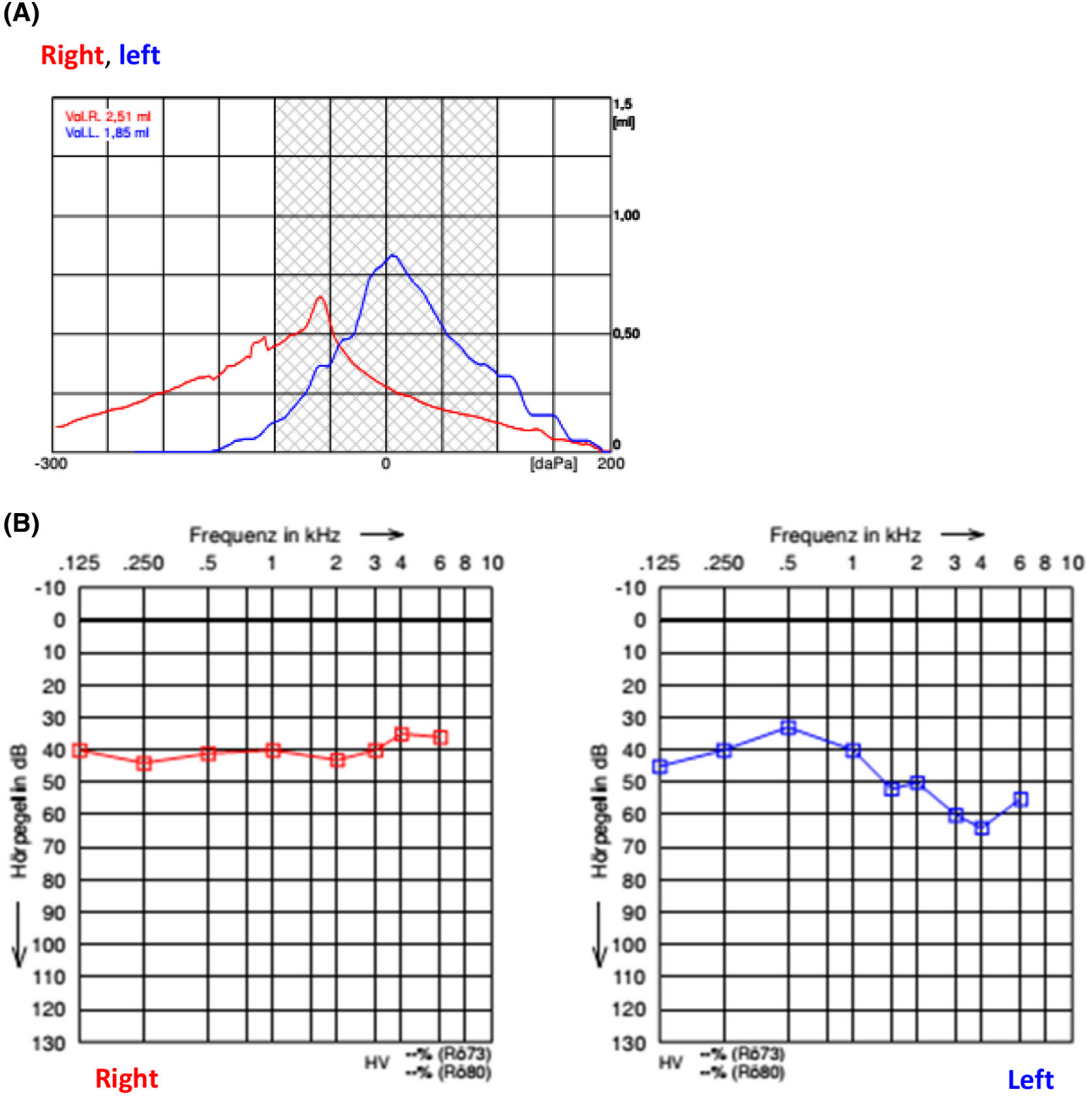
(A) Tympanometry of both ears. (B) Audiometry on the right ear using the CI and the left ear with the hearing aid. CI, cochlear implant.

At the age of 6 years, the follow‐up showed the following results: the right and left tympanic membrane was intact, tympanometry showed a well‐ventilated middle ear on both sides (Figure 6A). The audiometry on the right ear using the CI showed hearing level thresholds ranging between 30 and 40 dB at 0,125–6 KHz. The left ear with the hearing aid alone had hearing thresholds between 30–45 dB at 0,125–1 KHz and 50–62 dB at 1.5–8 KHz frequencies (Figure 6B). Speech assessment showed delays in all dimensions with heavily restricted speech comprehension, severely restricted vocabulary, moderately delayed morpho‐syntactic competence, and moderate phonological and articulation disorder. The parents reported good social competencies and inclusion.

## 5 DISCUSSION

This report describes the rare case of a right‐sided PLF with cochlear malformation, in an 8‐month‐old girl, successfully managed with surgical closure of the fistula and placement of a CI.

PLF is an abnormal communication between the perilymphatic space of the inner ear and the middle ear and/or mastoid. It is located in the thin membrane of the oval or round window, fractured bony labyrinth, microfissures, or anomalous stapedial foot plate. It can occur due to congenital causes such as ear malformation, can be acquired through head trauma, chronic inflammation, otic capsule dehiscence, or other iatrogenic causes. In some cases, the underlying cause remains unidentified. A child with such a congenital CSF fistula is estimated to have a 4%–50% risk of purulent or recurrent bacterial meningitis. In such cases, prophylactic antibiotics have not shown any significant preventive benefit. As bacterial meningitis is a life‐threatening disease with high mortality and morbidity, immediate surgical intervention is necessary.^3^, ^4^, ^5^

The diagnosis of PLF is a challenging one. It is accompanied by clinical signs such as hearing impairment, aural fullness with vestibular symptoms, in patients with a known history of IEM or head trauma or surgery. Otoscopy should be performed to detect any middle ear effusion containing CSF biomarkers. Radiography and explorative tympanotomy should also be performed to arrive at a comprehensive and definite diagnosis.^9^, ^10^, ^11^

In the case presented here, after surgery for middle ear effusion, CSF biomarkers were detected in the fluid of the middle ear. This warranted immediate surgical intervention. The perilymphatic leak could be identified under the stapedial footplate during surgery and was closed successfully.

IEMs are usually diagnosed by high resolution computed tomography (CT) and MRI.^3^, ^9^ In this case, the MRI revealed IEM in both ears with a CCD in the right ear and an IP‐1 malformation in the left ear. The IP‐1 malformation is also known as cystic cochleovestibular malformation, in which the modiolus and interscalar septa are lacking. In IP‐1 malformation, most children have severe to profound hearing loss.^12^, ^13^, ^14^, ^15^ CCD is the second most common IEM, in which the cochlea and vestibule are confluent forming one cystic cavity. In most children with CCD, no residual hearing has been reported.^9^ In the case presented here, the IEM was located in between the CCD and IP‐1; consequently, no hearing could be detected on the right side, however, a hearing threshold ranging between 70 and 80 dB was reported on the left side.

The key clinical management objectives of this case were to treat the profound hearing loss as well as closure of the perilymphatic leakage in the right ear. A clear CSF leakage coming from the stapedial region was identified as a PLF, which was surgically obliterated and the CI could be placed in the same surgical procedure, as the fistula could be sealed without covering the insertion positions for the CI. Different possible surgical approaches have been reported in the published literature, for closure of such fistulas, depending on their specific positions.^4^, ^5^, ^15^ An explorative tympanotomy is the only confirmatory diagnostic tool to detect a PLF, even if a high‐resolution CT scan of the temporal bones does not show any anomalies.^16^ Vlaski reported the case of a child with recurrent meningitis, diagnosed only intraoperatively, with a PLF of the stapedial footplate.^17^

The hearing improvement after CI is reported to be related to the number, distribution, and function of residual spiral ganglion cells as well as the placement of the electrodes in the cochlear cavity.^9^, ^18^ In our case, the post‐surgical CT scan confirmed appropriate positioning of the electrodes. Jesper Bille et al. concluded that CI showed similar long‐term outcomes in children with or without cochlear malformation.^19^ Isaiah et al. stated that CI is a safe and effective treatment in children with IEM, however, it shows poor speech outcomes.^20^ Zhang et al demonstrated that children with CCD who received a CI showed significant audiological development. However, their CI‐related outcomes were inferior as compared to children without CCD.^21^

Past evidence favors the use of CI in patients with residual hearing. In this case, residual hearing could not be detected in the affected ear, on ABR measurements, most likely due to increased hearing loss caused by the IEM.^22^ Our decision to opt for a CI in this case was driven by the fact that the child clearly suffered from bilateral hearing loss and could have significant speech and developmental delays, if remedial measures were not implemented at the right time. Moreover, after meningitis, implantation should be performed quickly because of the risk of inner ear obliteration. Besides, past evidence has clearly demonstrated that CI is safe and efficacious in children under the age of 12 months.^23^ Hence, we proceeded with the CI, despite the absence of residual hearing.

Regular audiometric examinations showed a restricted hearing outcome with CI, eliciting a response between 50 and 65 dB. Speech and language assessment at the age of six, revealed moderate to strong developmental delay. These outcomes are in congruence with the findings of Zhang et al, that highlight poorer CI‐related outcomes in children with a CCD as compared to those without it.^21^

We concur with past evidence that it is tough to predict whether a child with a CI fitted before 12 months of age, will achieve the same hearing and developmental ability as his normal counterparts. We also strongly believe that an early CI can definitely help the child achieve his or her maximum hearing and developmental potential, while minimizing associated risks. The developmental delay seen in our patient can most likely be attributed to the IEM.^23^

In the years following the surgery, there was no sign of CSF leakage or meningitis. The parents were informed and instructed to seek immediate specialized medical consultation, in case they noticed any clear rhinorrhea or middle ear effusion, without symptoms of infection.

## 6 CONCLUSION

Cochlear malformation accompanied by a perilymphatic leakage presents a challenging clinical scenario, especially due to the impending risk of bacterial meningitis, and thus warrants immediate surgical closure of the PLF. Wherever possible, the feasibility of a CI should be explored in such cases and a CI should be placed for treatment of hearing loss. Audiological and speech outcomes may vary with the use of the CI, especially in cases of IEM. However, an early CI coupled with timely PLF closure can help children with profound hearing loss, in achieving their maximum hearing and developmental potential, in the long run. Meticulous long‐term follow‐up for monitoring of hearing status, along with speech, language, and audiological training, play an essential part in improving the overall prognosis.

## AUTHOR CONTRIBUTIONS

**Hanan Almuzaini:** Conceptualization; investigation; methodology; writing – original draft. **J. Müller:** Conceptualization; project administration; supervision; validation; writing – review and editing. **Flatz Wilhelm:** Data curation; methodology; resources; writing – review and editing. **D. Polterauer:** Data curation; methodology; resources; validation; writing – review and editing. **M. Schuster:** Data curation; investigation; resources; visualization; writing – review and editing.

## FUNDING INFORMATION

This research received no specific grant from any funding Agency in the public, commercial, or not‐for‐profit sectors.

## CONFLICT OF INTEREST STATEMENT

All authors declare that they have no competing interests.

## CONSENT

Written informed consent was obtained from the patient's parents to publish this report in accordance with the journal's patient consent policy.

## Data Availability

The data that support the findings of this study are available from the corresponding author upon reasonable request.
